# The Role of 18F-Flortaucipir (AV-1451) in the Diagnosis of Neurodegenerative Disorders

**DOI:** 10.7759/cureus.16644

**Published:** 2021-07-26

**Authors:** Saswata Roy, Dipanjan Banerjee, Indrajit Chatterjee, Deepika Natarajan, Christopher Joy Mathew

**Affiliations:** 1 General Medicine, Musgrove Park Hospital, Taunton, GBR; 2 Internal Medicine, East Sussex Healthcare NHS Trust, Hastings, GBR; 3 Neuroscience, California Institute of Behavioral Neurosciences & Psychology, Fairfield, USA; 4 General Psychiatry, Wishaw University Hospital, Hamilton, GBR; 5 General Surgery, North Cumbria Integrated Care (NCIC), Carlisle, GBR

**Keywords:** 18f-av-1451, alzheimer's disease, frontotemporal dementia, dementia with lewy bodies, parkinson's disease, tau

## Abstract

Tau protein plays a vital role in maintaining the structural and functional integrity of the nervous system; however, hyperphosphorylation or abnormal phosphorylation of tau protein plays an essential role in the pathogenesis of several neurodegenerative disorders. The development of radioligand such as the 18F-flortaucipir (AV-1451) has provided us with the opportunity to assess the underlying tau pathology in various etiologies of dementia. For the purpose of this article, we aimed to evaluate the utility of 18F-AV-1451 in the differential diagnosis of various neurodegenerative disorders. We used PubMed to look for the latest, peer-reviewed, and informative articles. The scope of discussion included the role of 18F-AV-1451 positron emission tomography (PET) to aid in the diagnosis of Alzheimer’s disease (AD), frontotemporal dementia (FTD), dementia with Lewy bodies (DLB), and Parkinson’s disease with dementia (PDD). We also discussed if the tau burden identified by neuroimaging correlated well with the clinical severity and identified the various challenges of 18F-AV-1451 PET. We concluded that although the role of 18F-AV-1451 seems promising in the neuroimaging of AD, the benefit appears uncertain when it comes to the non-Alzheimer’s tauopathies. More research is required to identify the off-target binding sites of 18F-AV-1451 to determine its clinical utility in the future.

## Introduction and background

“None of us wants to be reminded that dementia is random, relentless, and frighteningly common.”

- Laurie Graham

Worldwide, around 50 million people are affected with dementia, with 10 million new diagnoses every year. This number is projected to reach 82 million in 2030 and 152 million by 2050, a 204% rise [1]. Etiologies of dementia are quite diverse, Alzheimer’s disease (AD) being the commonest one [2,3]. This is followed by vascular dementia (VaD), which is considered the second most common cause of dementia [4]. Other causes of dementia include frontotemporal dementia (FTD), dementia with Lewy bodies (DLB), Parkinson’s disease with dementia (PDD) [5,6]. In both the central and peripheral nervous system, microtubule assembly (MT) is promoted by a microtubule assembly protein (MAP), also known as Tau protein. By playing a pivotal role in MT assembly and stabilization, Tau protein helps to maintain the structural and functional integrity of the nervous system [7]. However, abnormal phosphorylation of tau protein results in filamentous deposits in neurons and glial cells, contributing to the pathogenesis of various neurodegenerative disorders [7]. Such lesions have been described in AD, and other neurodegenerative disorders popularly referred to as tauopathies [8]. 18F-flortaucipir (AV-1451) is a radiotracer widely used in positron emission tomography (PET) which shows high affinity towards tau protein deposits and used in neuroimaging of neurodegenerative disorders involving tau protein abnormalities [9]. In this review, we aim to briefly evaluate and summarize the role of 18F-AV-1451 PET imaging in the diagnosis of various common etiologies of dementia.

## Review

Method

For the purpose of this literature review, PubMed was searched extensively with appropriate keywords mentioned, to look for relevant articles. We included the studies conducted within the last eight years, specifically on humans. A total of 31 studies were included after considering the citations, year of publication, subject population, and statistical significance. Older studies or studies conducted on other species were not included. Data was collected ethically and legally.

Discussion

Tau Protein: A Brief Introduction

Tau protein is also known as microtubule assembly protein (MAP) which helps maintain the structural and functional integrity of neurons by promoting and stabilizing microtubules [10]. The gene responsible for the synthesis of Tau protein is located on chromosome 17 and contains 16 exons [7]. Out of these, exons 9-12 code for four microtubule-binding motifs, and alternative splicing of exon 10 leads to the formation of tau isoforms with three (3R) or four repeat (4R) domains [7]. In total, there are six tau isoforms present in the adult brain [7]. Posttranslational modification involving phosphorylation plays a significant role in diversifying the tau isoforms [11]. This process of phosphorylation is carefully controlled by the activity of tau kinase and phosphatase [11]. Disruption of this equilibrium leads to abnormal or hyperphosphorylation of tau protein resulting in the accumulation of tau protein aggregates which play an important role in the pathogenesis of various etiologies of dementia [11].

Alzheimer’s Disease: Dual Pathogenesis

Alzheimer’s disease (AD) is the commonest form of dementia, with 6.2 million affected in the USA, a number that is projected to increase to 13.8 million by 2060 [5]. The underlying pathology in AD involves the formation of extracellular amyloid plaques, and intracellular neurofibrillary lesions (NFL) made up of abnormally phosphorylated tau protein [12]. A study done on AD and progressive supranuclear palsy (PSP) patients using 18F-AV-1451 PET imaging established increased 18F-AV-1451 retention in the hippocampus and frontal, occipital, and temporoparietal cortices of AD patients when compared to PSP subjects [13]. The PSP group, on the other hand, showed increased retention in the region of the midbrain when compared to AD patients [13]. A study comparing antemortem PET findings and post mortem autopsy findings found out that there was a good correlation between areas of 18F-AV-1451 retention and neurofibrillary lesion distribution in AD patients [14]. The group with non-Alzheimer’s tauopathies and non-tau pathologies showed lower retention of 18F-AV-1451 compared to the AD group but higher when compared to the control population [14]. While analyzing the Braak stages, it was also established that patients with advanced Braak stages showed higher tracer retention while the patients with lower Braak stages failed to show increased uptake compared to the control population [14]. Comparing tau imaging with 18F-AV-1451 PET and beta-amyloid imaging with PiB-PET, it has been suggested that the regional distribution of 18F-AV-1451 retention shows a stronger association with different clinical variants of AD than amyloid distribution [15]. For example, patients with posterior cortical atrophy (PCA) showed higher 18F-AV-1451 uptake in the posterior fossa while PiB binding was elevated across the whole neocortex [15]. Similarly, patients with predominant memory issues showed higher 18F-AV-1451 retention in the medial temporal and lateral temporoparietal areas [15]. Similar findings were also shared by other studies [16]. The relevant studies are summarized below in Table 1.

**Table 1:** Summary of findings in Alzheimer's disease AD: Alzheimer's disease; FTD: frontotemporal dementia; PSP: progressive supranuclear palsy; HC: healthy controls; PET: positron emission tomography; FDG-PET: fluorine-18-fluorodeoxyglucose positron emission imaging; PiB-PET: Pittsburgh compound B positron emission tomography.

Author	Imaging modality	Subjects	Summary of findings
Passamonti et al. [13]	18F-AV-1451 PET	15 AD, 19 PSP, 13 HC	AD group showed higher retention in the hippocampus and frontal, occipital, and temporoparietal cortices, while PSP patients showed increased retention in the region of the midbrain.
Soleimani-Meigooni et al. [14]	18F-AV-1451 PET	8 AD, 9 non-Alzheimer tauopathies and 3 non-tau FTD	Good correlation between areas of 18F-AV-1451 retention and neurofibrillary lesion distribution in post-mortem AD patients, also higher amount of retention was seen in more advanced Braak stages.
Ossenkoppele et al. [15]	18F-AV-1451 PET, PiB-PET, FDG-PET	20 AD, 15 HC	Compared to other radiotracers, the regional distribution of 18F-AV-1451 retention correlates well with the different regions of the brain affected in various variants of AD.
Hoenig et al. [16]	18F-AV-1451 PET	22 AD, 26 HC	Higher uptake was associated with higher Braak stages, regional uptake correlated well with functional networks involved.

Frontotemporal Dementia (FTD)

A familial form of FTD was identified in 1994 linked to the MAPT gene on chromosome 17, which was later named frontotemporal dementia, and parkinsonism linked to chromosome 17 (FTDP-17) due to its similar clinical, neurological, and genetic profile [7,17]. A study conducted on 45 patients of FTD including various variants and 53 control subjects showed the following: nonfluent variant primary progressive aphasia (nfvPPA) patients showed increased 18F-AV-1451 binding in the left inferior frontal gyrus (asymmetrically, more than right hemisphere), patients with corticobasal syndrome (CBS) showed raised binding in frontal white matter [18]. Increased 18F-AV-1451 retention was noticed in bilateral temporal lobes in carriers of MAPT mutation, and frontotemporal binding was observed in 50% of the behavioral variant frontotemporal dementia (bvFTD) patients [18]. Another study by Bevan-Jones et al. showed markedly raised non-displaceable binding potential (BPND) of 18F-AV-1451, especially in the anterior temporal lobes and ventral anterior cingulate cortex of patients with MAPT mutations compared to the healthy control subjects [19]. Smith et al. observed increased 18F-AV-1451 retention in the regions of the hippocampus and adjacent temporal lobe in patients with MAPT mutations [20]. This study also observed the extent of 18F-AV-1451 retention to be associated with the clinical severity of the disease observed [20]. They also performed a post-mortem analysis in a patient and found out that the areas which showed increased antemortem 18F-AV-1451 binding also tested positive for tau pathologies post-mortem and even tested negative amyloid staining [20]. Elevated BPND was observed in patients with semantic dementia in the regions of the temporal cortex, insula, and fusiform gyrus, which correlates well with the regions known to be affected in this variant [21]. This study displayed 86% sensitivity and 100% specificity when differentiating between patients of semantic dementia and the control subjects [21]. The findings of these studies are summarized in Table 2.

**Table 2:** Summary of findings in frontotemporal dementia AD: Alzheimer's disease; FTD: frontotemporal dementia; PSP: progressive supranuclear palsy; HC: healthy controls; PET: positron emission tomography; FDG-PET: fluorine-18-fluorodeoxyglucose positron emission imaging; PiB-PET: Pittsburgh compound B positron emission tomography; bvFTD: behavioral variant of frontotemporal dementia; nfvPPA: nonfluent variant primary progressive aphasia; CBS: corticobasal syndrome; svPPA: semantic variant primary progressive aphasia; BPND: non-displaceable binding potential; MAPT: microtubule assembly protein tau; C9ORF72: chromosome 9 open reading frame 72; GRN: progranulin gene.

Author	Imaging modality	Subjects	Summary of findings
Tsai et al. [18]	18F-AV-1451 PET, PiB-PET, MRI	45 FTD (11 nfvPPA, 10 CBS, 10 bvFTD, 2 svPPA, 6 MAPT mutants, 5 C9ORF72 mutants, 1 GRN mutant), 53 HC	nfvPPA: Increased AV-1451 binding in the left inferior frontal gyrus compared to the right hemisphere, CBS: Higher 18F-AV-1451 binding in the frontal white matter, bvFTD: Five out of 10 patients showed increased 18F-AV-1451 retention in the frontotemporal region, MAPT carriers: Higher 18F-AV-1451 binding in the bilateral temporal lobes compared to controls.
Bevan Jones et al. [19]	18F-AV-1451 PET	A case of familial FTD with MAPT mutation, 12 HC	Significantly increased BPND in MAPT carrier especially in the anterior temporal lobes and ventral anterior cingulate cortex compared to the control subjects.
Smith et al. [20]	18F-AV-1451 PET, 18F-flutemetamol PET, FDG-PET	3 MAPT mutants, 4 HC, 5 AD	Raised 18F-AV-1451 binding in the regions of the hippocampus and adjacent temporal lobe in MAPT mutants, widespread retention was seen in the patient with higher clinical severity.
Bevan-Jones et al. [21]	PET using 11C-PK-11195, 18F-AV-1451	31 FTD (10 bvFTD, 11 svPPA, 10 nfvPPA), 29 HC	Markedly raised BPND in semantic dementia in the regions of the temporal cortex, insula, and fusiform gyrus, also found a good correlation between neuroinflammation and tau retention.

Dementia with Lewy Bodies (DLB)

DLB is characterized by a significantly lower uptake of 18F-AV-1451, especially in the medial temporal lobe compared to AD [22]. However, increased retention of 18F-AV-1451 was seen in the posterior temporoparietal and occipital cortex of the DLB group compared to the control group which correlated well with the PiB retention, suggesting an atypical pattern of tau pathology in DLB [22]. Another study conducted by Gomperts et al. on DLB, PDD, and PD without dementia patients showed increased 18F-AV-1451 retention in both DLB and PDD patients compared to the control groups and PD without dementia patients [23]. Although the tau burden was much lower than AD, the topographical distribution of high tracer retention mimicked that of the AD [23]. The extent of 18F-AV-1451 uptake was also found to correlate well with the clinical severity of cognitive dysfunction as measured by mini-mental state examination (MMSE) [23,24]. Increased parietal 18F-AV-1451 retention has also been recorded in DLB patients and this was linked to the abnormality of executive functions in these patients [25]. These findings are summarized below in Table 3.

**Table 3:** Summary of findings in dementia with Lewy bodies AD: Alzheimer's disease; PD: Parkinson's disease; PDD: Parkinson's disease with dementia; DLB: dementia with Lewy bodies; HC: healthy controls; PET: positron emission tomography; FDG-PET: fluorine-18-fluorodeoxyglucose positron emission imaging; PiB-PET: Pittsburgh compound B positron emission tomography; MRI: magnetic resonance imaging.

Author	Imaging modality	Subjects	Summary of findings
Kantarci et al. [22]	18F-AV-1451, PiB PET	19 DLB, 19 AD, 95 HC	18F-AV-1451 retention in DLB is lesser compared to AD, but higher in the posterior temporoparietal and occipital cortex compared to the control group.
Gomperts et al. [23]	18F-AV-1451, PiB PET	7 DLB, 8 PDD, 9 PD without dementia, 29 HC	Markedly raised 18F-AV-1451 retention in both DLB and PDD compared to the healthy controls, increased tau burden correlates well with the clinical severity.
Smith et al. [25]	18F-AV-1451 PET, MRI	6 DLB, 18 PDD, 11 PD without dementia, 44 HC	Raised parietal 18F-AV-1451 binding in DLB patients, which was linked to abnormality in executive functions in patients.

Parkinson's Disease (PD)

PDD patients have shown raised 18F-AV-1451 binding in the inferior temporal gyrus and precuneus compared to the control population suggesting a role of tau pathology [23]. The degree of tau burden, although remarkably less than that of AD, has also been shown to correlate well with the clinical severity of the disease [23]. Another study demonstrated reduced 18F-AV-1451 retention in the substantia nigra in PDD [25]. This was also found to be associated with worsening motor functions [25]. The total nigral 18F-AV-1451 volume of distribution was measured to be 30% lower in patients with idiopathic PD compared to the healthy control population [26]. However, no association has been found between the side with dominant symptoms and the contralateral nigral volume of distribution [26]. Another study conducted on PSP and PD patients showed a remarkably decreased nigral standardized uptake value ratio (SUVr) compared to the healthy controls [27]. However, no remarkable difference was observed in terms of nigral SUVr between PD and PSP groups [27]. Researchers observed raised subcortical 18F-AV-1451 uptake in PD and PSP patients but did not find any correlation with the severity of motor symptoms [14,28]. Tau pathology has also been found to be uncommon in patients with PD and mild cognitive impairment [29]. The findings of these studies are summarized below in Table 4.

**Table 4:** Summary of findings in Parkinson's disease PD: Parkinson's disease; PDD: Parkinson's disease with dementia; DLB: dementia with Lewy bodies; PSP: progressive supranuclear palsy; HC: healthy controls; PET: positron emission tomography; FDG-PET: fluorine-18-fluorodeoxyglucose positron emission imaging; PiB-PET: Pittsburgh compound B positron emission tomography; MRI: magnetic resonance imaging; [123I]FP- CIT-SPECT: Iodine 123-radiolabeled 2β-carbomethoxy-3β-(4-iodophenyl)-N-(3-fluoropropyl) nortropane single-photon emission tomography.

Author	Imaging modality	Subjects	Summary of findings
Gomperts et al. [23]	18F-AV-1451, PiB PET	7 DLB, 8 PDD, 9 PD without dementia, 29 HC	Although less than AD, 18F-AV-1451 retention is higher in PDD group compared to the control group.
Smith et al. [25]	18F-AV-1451 PET, MRI	6 DLB, 18 PDD, 11 PD without dementia, 44 HC	Reduced 18F-AV-1451 retention was seen in the substantia nigra of PDD patients.
Hansen et al. [26]	(123)I-FP-CIT SPECT, 18F-AV-1451 PET	17 idiopathic PD, 16 HC	The nigral 18F-AV-1451 volume of distribution was significantly reduced in idiopathic PD patients than in the control group.
Coakeley et al. [27]	18F-AV-1451 PET	12 patients including PD and PSP, 15 HC	Significantly decreased nigral 18F-AV-1451 uptake in both PD and PSP patients compared to the control groups, no remarkable difference observed between PD and PSP patients.
Cho et al. [28]	18F-AV-1451 PET	14 PSP, 15 PD, 15 HC	Raised sub-cortical 18F-AV-1451 retention was seen in PD and PSP patients and reduced nigral 18F-AV-1451 volume of distribution was noticed in PD patients.

Pitfalls of 18F-AV-1451

18F-AV-1451, although showing promise in detecting advanced stages of AD with higher Braak stages, such results were not replicated in patients with earlier Braak stages compared to the control population [14]. This might indicate reduced affinity of 18F-AV-1451 towards less mature tau formations [30]. The role of 18F-AV-1451 in detecting non-Alzheimer’s tauopathies has been doubted by several researchers [14,21]. This has been partly explained by the finding that 18F-AV-1451 showed more affinity towards paired helical filaments rather than straight filaments found in abundance in non-Alzheimer’s tauopathies [31]. Histologically AD is characterized by mixed 3R and 4R tau deposits, which are more actively recognized by 18F-AV-1451 than disorders that are mainly known to have an accumulation of either 3R or 4R [30]. The inconsistency of findings between in-vivo and post-mortem subjects also popularized the idea of off-target binding for 18F-AV-1451 [21]. Such off-target sites were observed by studies in vessels, melanin, and neuromelanin-containing tissues [30,31]. Researchers agree that more knowledge and recognition of such off-target sites may play a vital role in determining the clinical utility of 18F-AV-1451 [30].

## Conclusions

After a detailed review, we conclude that 18F-AV-1451 shows promise in demonstrating the underlying tau pathology in AD, particularly in advanced stages where it correlates well with higher Braak stages. However, its role in non-Alzheimer’s tauopathies remains uncertain due to the observation that it shows less affinity towards straight filaments, which plays an important role in such neurodegenerative disorders. 18F-AV-1451 may be one of the first biomarkers to provide neuroimaging of the nigral pathology in PD, but more research is required both in-vivo and post-mortem for the recognition of several off-target binding sites, which will also play a significant role in assessing the clinical utility of 18F-AV-1451 PET.
